# Acute Impacts of Different Types of Exercise on Circulating α-Klotho Protein Levels

**DOI:** 10.3389/fphys.2021.716473

**Published:** 2021-09-01

**Authors:** Tamara Iturriaga, Thomas Yvert, Isabel M. Sanchez-Lorente, Ignacio Diez-Vega, Valentin E. Fernandez-Elias, Lara Sanchez-Barroso, Diego Dominguez-Balmaseda, Mar Larrosa, Margarita Perez-Ruiz, Catalina Santiago

**Affiliations:** ^1^Faculty of Physical Activity, Sport Sciences and Physiotherapy, Universidad Europea de Madrid, Madrid, Spain; ^2^Departamento de Enfermería y Fisioterapia, Facultad de Ciencias de la salud, Universidad de Leon, Ponferrada, Spain; ^3^Facultad de Enfermería, Fisioterapia y Podología, Universidad Complutense de Madrid, Madrid, Spain; ^4^Faculty of Biomedical and Health Sciences, Universidad Europea de Madrid, Madrid, Spain; ^5^Servicio de Medicina Física y Rehabilitación, Hospital General Universitario Gregorio Marañón, Instituto de Investigación Sanitaria Gregorio Marañón, Madrid, Spain

**Keywords:** klotho, biomarker, physical condition, endurance exercise, strength exercise

## Abstract

**Introduction:** Elevated plasma α-klotho (αKl) protects against several ageing phenotypes and has been proposed as a biomarker of a good prognosis for different diseases. The beneficial health effects of elevated plasma levels of soluble αKl (SαKl) have been likened to the positive effects of exercise on ageing and chronic disease progression. It has also been established that molecular responses and adaptations differ according to exercise dose. The aim of this study is to compare the acute SαKl response to different exercise interventions, cardiorespiratory, and strength exercise in healthy, physically active men and to examine the behavior of SαKl 72h after acute strength exercise.

**Methods:** In this quasi-experimental study, plasma SαKl was measured before and after a cardiorespiratory exercise session (CR) in 43 men, and strength exercise session (ST) in 39 men. The behavior of SαKl was also examined 24, 48, and 72h after ST.

**Results:** Significant differences (time×group) were detected in SαKl levels (*p*=0.001; *d*=0.86) between CR and ST. After the ST intervention, SαKl behavior varied significantly (*p*=0.009; *d*=0.663) in that levels dropped between pre- and post-exercises (*p*=0.025; *d*=0.756) and were also significantly higher compared to pre ST values at 24h (*p*=0.033; *d*=0.717) and at 48h (*p*=0.015; *d*=0.827).

**Conclusions:** SαKl levels increased in response to a single bout of cardiorespiratory exercise; while they decreased immediately after strength exercise, levels were elevated after 24h indicating different klotho protein responses to different forms of exercise.

## Introduction

The αKlotho (αKl) gene was discovered in 1997 by Kuro-o et al. (1997). In a study in mice, these authors found that a mutation in the αKl gene caused typical symptoms of rapid ageing such as sarcopenia, low bone mineral density, and skin atrophy (Kuro-o et al., 1997). More recent studies have focused on the molecular mechanisms of action of this protein (Xu and Sun, 2015), and its usefulness as a prognostic biomarker for several diseases including cardiovascular disease in patients with chronic kidney disease (Neyra and Hu, 2016), lung diseases (Kureya et al., 2016; Pako et al., 2017), cancer (Kuro-o, 2019), and some autoimmune diseases (Guzmán-Silahua et al., 2017). These studies have been consistent in revealing that the lower levels of αKl are linked to a worse disease prognosis. Low αKl levels have been also related to premature ageing (Felipe Salech et al., 2012), systemic inflammation (Krick et al., 2018), endothelial cell damage (Matsubara et al., 2014), impaired cell regeneration and proliferation (Kuro-o, 2009; Welc et al., 2020), oxidative stress (Yamamoto et al., 2005), and abnormal bone mineral metabolism (Razzaque, 2012).

Exercise is an effective tool to treat and prevent many causes of morbidity and mortality associated with ageing (Cordero et al., 2014) and chronic diseases (Ostman et al., 2017; Paneroni et al., 2017; Qiu et al., 2017). Depending on the training modality and dose, the adaptations produced vary widely (Egan and Zierath, 2013). Recently, exercise has been proposed as a good strategy to improve blood levels of SαKl as it seems to have increasing effects on muscle mass and decreasing ones on fat mass (Amaro-Gahete et al., 2019). Other studies have shown a direct relationship between physical fitness, as measured through maximal oxygen consumption, and SαKl levels, such that the plasma levels of protein are higher in both young and older people who are physically fitter (Matsubara et al., 2014; Amaro-Gahete et al., 2018). Avin et al. even highlighted the similarity between the beneficial effects of high SαKl levels and exercise, namely improved resistance to oxidative stress, along with increased tissue regeneration, bone mass, angiogenesis, and endothelial cell protection (Avin et al., 2014). Changes in plasma αKl levels could be explained by the role of the klotho gene in molecular signaling pathways in muscle cells, acutely regulating the removal of reactive oxygen species (ROS) and chronically regulating the inflammatory process (Yamamoto et al., 2005; Li et al., 2016; Ji et al., 2018), consistent with its proposed mechanisms of action in anti-ageing effects. We also propose that exercise, dosage, intensity, and probably modality may be important in the response of SαKl to exercise. Preliminary results have indicated that an acute session of moderate-high intensity cardiorespiratory exercise (>70% VO_2max_) produces a sustained increase in blood levels of SαKl (Mostafidi et al., 2016; Santos-Dias et al., 2017), possibly lasting up to 7days after the exercise session (Tan et al., 2018). In addition, several weeks of cardiorespiratory training seems to affect plasma SαKl concentrations irrespective of training intensity (low, moderate, or high; Avin et al., 2014; Matsubara et al., 2014; Saghiv et al., 2019). In contrast, an acute low-intensity intervention (40–55% VO_2max_) was found to elicit no change in this biomarker in both young and older individuals (Avin et al., 2014).

Most investigations that have addressed the effects of exercise on levels of this protein have mainly focused on cardiorespiratory exercise. These studies have shown that individuals who regularly practice any activity with a high cardiorespiratory component have greater plasma levels of SαKl (Saghiv et al., 2015a; Mostafidi et al., 2016). However, few studies have examined the effects of strength training on SαKl (Semba et al., 2012, 2015; Saghiv et al., 2017a) and the impacts of acute vs. chronic strength training interventions have not yet been explored. To date, however, there is a sufficient knowledge of molecular mechanisms triggered by strength training to suggest that this form of exercise could have a positive effect on SαKl. For example, it is known that strength exercise induces the release of insulin-like growth factor 1 (IGF-1; Adams, 1998) in the muscle cell, where it induces protein synthesis producing adaptations such as muscle fiber growth (Egan and Zierath, 2013). To elicit these adaptations, exercise programs have been designed to prioritize eccentric efforts able to induce controlled muscle damage, such as plyometric training, thus triggering molecular pathways that lead to adaptations that are beneficial for both general and muscle health. Further, as does cardiorespiratory exercise, this metabolic activity generates ROS, but at a later time point (24–48h after a training session). The controlled muscular damage that gives rise to ROS in the first hours of strength training is necessary for protein metabolism (Egan and Zierath, 2013). The buildup of ROS, before or after depending on the type of trigger produces sufficient oxidative stress to cause the deterioration of muscle cell. It is, at this point, that the role of SαKl protein seems critical. There are two possible mechanisms of cell resistance to oxidative stress mediated by SαKl: the first would be IGF-1 receptor inhibition diminishing ROS production and, the second, Akt inhibition, would prevent the phosphorylation of FOXO (*Forkhead box O*) to activate the enzymes such as superoxide dismutase (SOD2; Kuro-o, 2009; Papaconstantinou, 2009; Daneshgar and Dai, 2020).

Given this described association between SαKl and exercise in molecular terms along with the beneficial effects of both factors on some chronic conditions, this study sought to compare the effects of cardiorespiratory and strength exercise on levels of this protein. While it seems that both types of exercise could be used as a tool to increase SαKl levels, the behavior of the protein after a strength intervention remains unknown. The aims of the present study were: (i) to compare the acute effects of cardiorespiratory vs. strength exercise on SαKl levels in healthy physically active men and (ii) to monitor SαKl levels over a 72-h period after a bout of strength exercise.

## Materials and Methods

### Design and Sample

**Figure 1:**
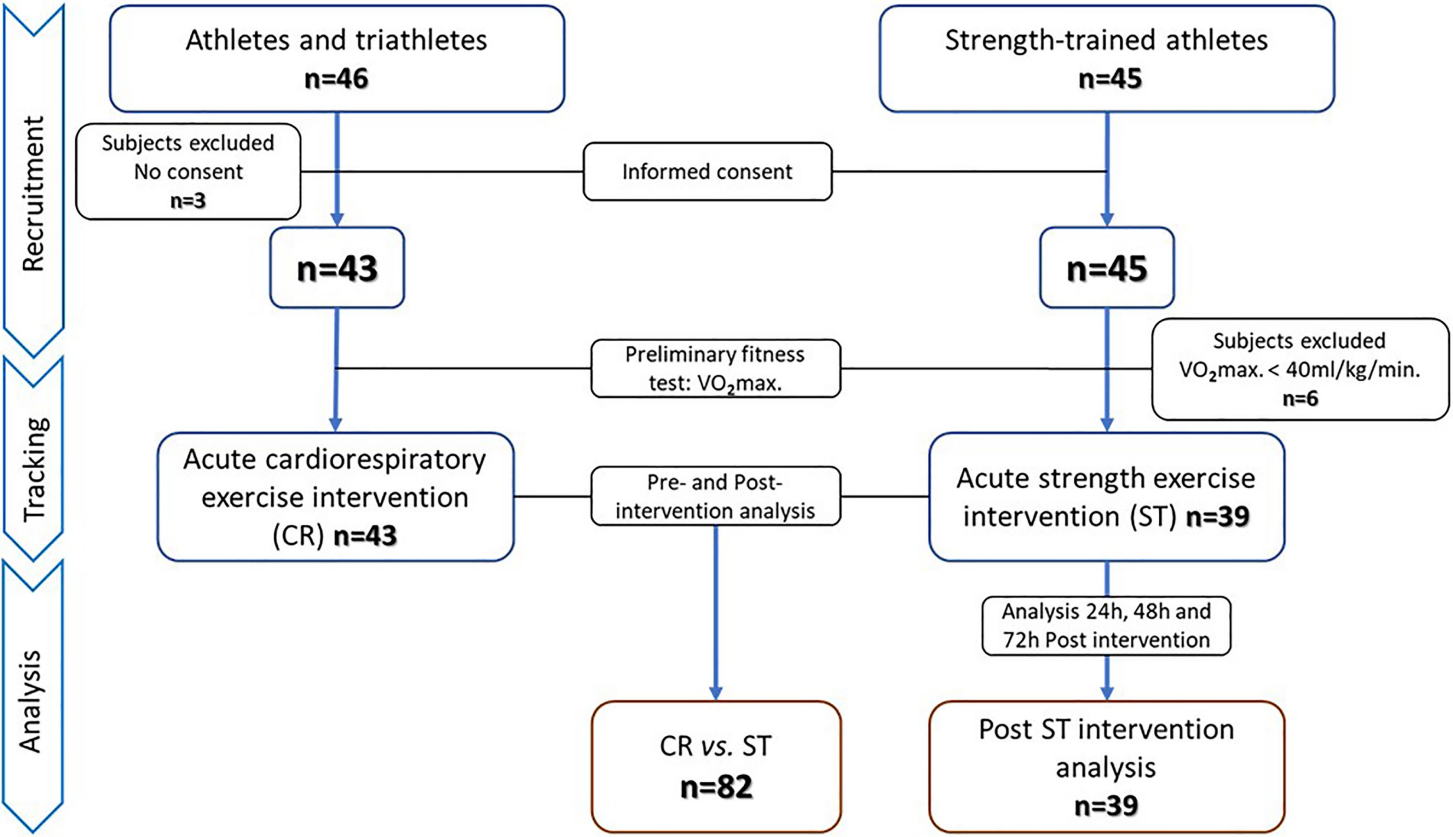
Flow chart detailing the recruitment, tracking, and analysis of the study participants.

Our first aim was addressed in a quasi-experimental study with pre–post measurements, and our second aim in a single-arm trial. The study protocol adhered to the “Ethical Guidelines of the Declaration of Helsinki,” as last amended in 2013, and was approved by the Regional Clinical Research Ethics Committee of the Community of Madrid (CEIm-R. Reference number: 47/764390.9/17). All participants gave their written informed consent. Through a non-probabilistic convenience sampling approach, two independent groups of physically active men who had training experience in different sports modalities were selected to establish two study groups: acute cardiorespiratory (CR) and acute strength (ST) exercise. Inclusion criteria were: (i) men aged 18–55years, (ii) non-smokers, (iii) cardiorespiratory or strength training at least three times a week for 6months or longer, and (iv) no musculoskeletal injuries sustained in the past 6months. Exclusion criteria were: (i) a fitness level below a maximal oxygen consumption (V0_2max_) of 40ml/kg/min, (ii) exercise intervention not completed and/or blood samples not collected, and (iii) no informed consent given. According to these inclusion/exclusion criteria, 46 men from athletics and triathlon clubs were selected for the CR group and 45 men from different sports centers whose training was mainly strength-based for the ST group. Three subjects in the CR group decided not to participate after reading the informed consent form. The remaining 43 men in this group underwent preliminary physical fitness and body composition tests. In all 43 subjects, V0_2max_ was above 40ml/kg/min, and these individuals were selected for the acute cardiorespiratory exercise intervention. All completed the intervention and provided all required pre- and post-intervention blood samples. All 45 subjects selected for the ST group decided, and they would participate in the study, and therefore, performed the pretest. Six of these individuals were excluded because of a V0_2max_ below 40ml/kg/min, leaving 39 subjects in the acute strength exercise group. All completed the intervention and sampling protocol. Subjects in this ST group were also scheduled for additional sampling at 24, 48, and 72h after the session to assess the behavior post-exercise of SαKl levels. The recruitment, tracking and analysis procedures are detailed in the flow chart (Figure 1).

### Study Variable

#### Plasma SαKl

A blood sample was collected from the antecubital vein into a separating gel vacuum tube, centrifuged, and stored at −80°C until analysis using an enzyme-linked immunosorbent assay (ELISA) kit (Human Soluble Test Kit α-Klotho Immuno-Biological Laboratories Co., Ltd., Japan). Assays were run in duplicate as double parallel tubes following the manufacturer’s recommendations. This procedure has an intra-assay coefficient of variation (CV) of 3.1% and an inter-assay CV of 6.9%, with a sensitivity of 6.15pg/ml (Yamazaki et al., 2010). The CV was ≤1.9% in all the samples.

### Interventions

**Figure 2:**
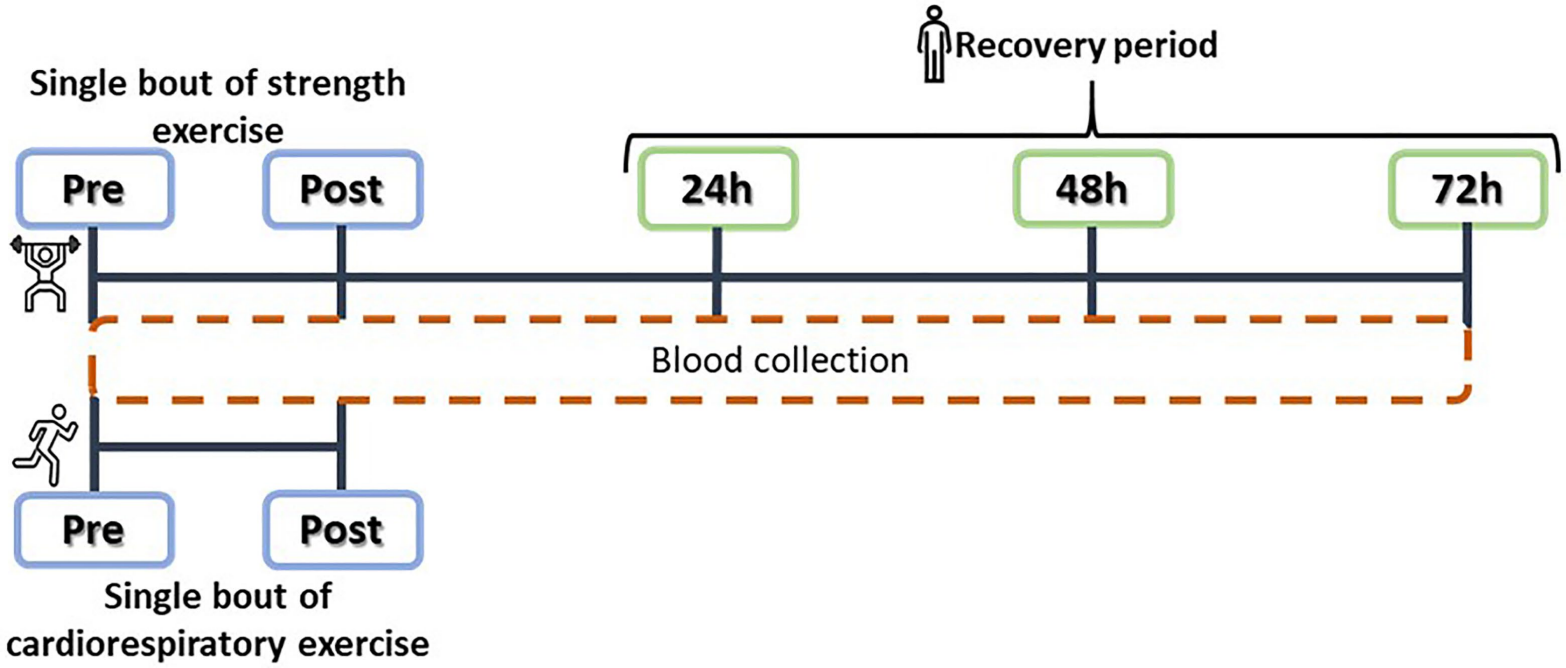
Experimental design: acute cardiorespiratory (CR) and strength training (ST) interventions, and plasma SαKI determinations.

The CR intervention consisted of a 10-min warm-up at 55% of estimated V0_2max_, a steady treadmill run at 75% of V0_2max_ lasting 30min, and a cool down of 10min of slow walking (Saghiv et al., 2015b). Blood samples were collected just before the start of the exercise intervention and just after the end of the intervention (Figure 2).

The main objective of the ST intervention was to induce controlled muscle damage through eccentric contractions at an intensity of >70% of the rate of perceived exertion (RPE) of each subject. The intervention consisted of a warm-up on a cycle ergometer for 10min followed by a set of plyometric exercises: 100 jumps from a 60cm box with dumbbells in each hand representing 10% of body weight; as soon as contact was made with the ground after each jump, the weights were dropped and a vertical jump was completed at maximum power. The session was structured as five sets of 20 jumps with 2min rest intervals between sets and 10s of rest between each jump and took approximately 1hour to complete (Howatson et al., 2012). Blood samples were collected at five time points: just before starting the exercise (Pre), just after finishing it (Post), and over the following 3days (24, 48, and 72h; Figure 2). No other form of exercise was performed over the blood sampling period and the blood samples were taken between 9 and 11AM for all the participants.

### Baseline Subject Characteristics

This information is highly measured and analyzed to establish baseline parameters for comparison between both groups.

The demographic and clinical data collected were age in years, hours per week of exercise, musculoskeletal injuries in the last 6months, and any pre-existing diseases.

#### Physical Fitness

This variable was recorded as VO_2max_ on a treadmill ergo-spirometer (analyzer: ULTIMA series, MEDGRAPHICS, Cardiorespiratory diagnostic; treadmill: Venus 200/75. h/p/cosmos. Nussdorf-Traunstein, Germany) in an incremental protocol consisting of 3min of warm-up at 7.7km/h, followed by an increase of 0.3km/h every 30s until exhaustion, applying maximality criteria according to the procedure described by Casajús et al. (2009).

#### Body Composition

Height and weight measured using a stadiometer and analogue scales (Ano Sayol SL, Barcelona, Spain, and Asimed T2, Barcelona, Spain, respectively). A Dual X-Ray Densitometry (DEXA) system (Hologic QDR Discovery, Bedford, MA, United States) was used for the 8-min “full-body” test (Plank, 2005). The variables recorded were: body mass index (BMI), body adipose index (BAI) as a percentage (%), appendicular skeletal muscle mass (ASM) in arms and legs as a whole, and visceral fat area (VFA) in square centimeters (cm^2^).

### Statistical Analysis

For a final study population of 82 subjects, descriptive statistics are provided as the mean, standard deviation and confidence interval (95% CI) of each variable, reflecting the characteristics of the sample. For comparisons between the two interventions, a mixed ANCOVA (2×2) was used with age and V0_2max_ as covariates, as these two factors may affect SαKl levels (Avin et al., 2014; Amaro-Gahete et al., 2018). *Post-hoc* comparisons were carried out with Bonferroni correction. In the ST group, SαKl behavior was examined through repeated-measures ANOVA, comparing differences (at Post, 24, 48, and 72h) with baseline levels (Pre) using *a priori* test of simple within-subject contrasts. The effect size was reported as Cohen’s *d* (*d*) and interpreted according to Cohen’s recommendations (Cohen, 1988). The accuracy of the SαKl values recorded was determined through CVs calculated for duplicates of each sample. All the statistical tests were performed using the package SPSS 20.1 for Windows. Significance was set at *p*<0.05.

## Results

Participants had similar demographic and physiological characteristics (Table 1). Baseline SαKl values were 1018.44±237.24pg/ml, and the mean CV between duplicates of all the samples was 1.74% (duplicate measures with a CV higher than 10% were not accepted).

**Table 1:** Characteristics of the study participants.

	Mean±SD			*p*
	Total (*n*=82)	CR (*n*=43)	ST (*n*=39)	
Age (years)	29.9±9	35.8±8.14	23.3±3.9	0.001
Weight (kg)	72.9±8.7	70.6±8.1	75.5±8.8	0.010
Height (cm)	176.8±6.8	175.4±6.2	178.3±7.2	0.051
BMI (kg/m^2^)	23.2±2.1	22.8±2.2	23.6±1.9	0.063
BAI (%)	18.7±3.5	19.1±4.1	18.4±2.6	0.070
VFA (cm^2^)	57.8±24	64.6±29.5	50.4±12.5	0.007
ASM (kg)	8.3±0.7	8.2±0.7	8.3±0.7	0.564
VO_2max_ (ml/kg/min)	56.9±5.2	58.2±4.9	55.6±5.1	0.023

Results shown as the mean and standard deviation (mean±SD). Significance set at *p*<0.05. BMI: body mass index; BAI: body adipose index; VFA: visceral fat area; ASM: appendicular skeletal muscle mass.

**Figure 3:**
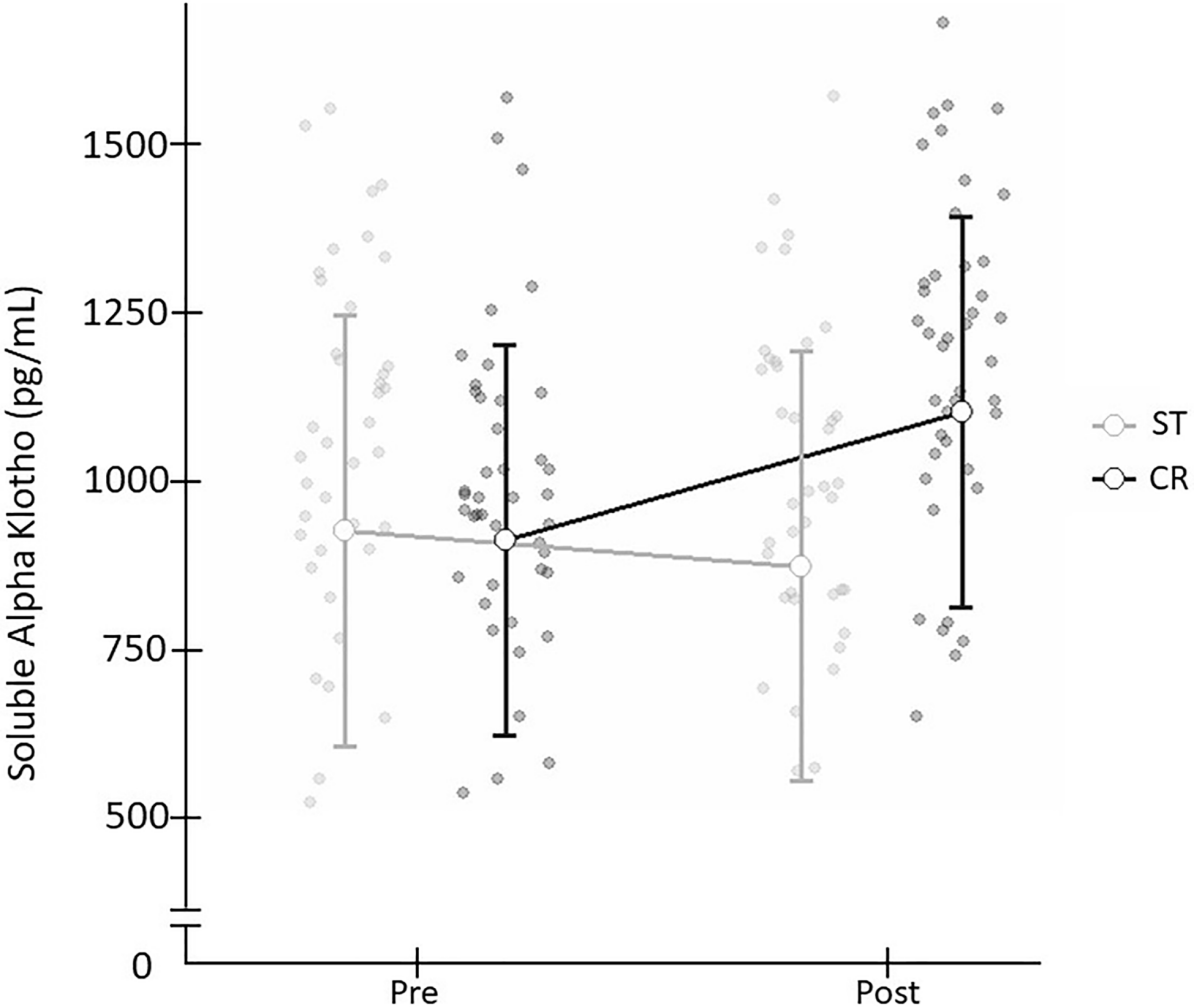
Plasma SαKI levels determined before and after the exercise intervention in the CR and ST groups.

When comparing the two exercise interventions, a significant time×group (*t*×*g*) interaction was found with a large effect size [F(1,78)=14.476; *p*=0.001; *d*=0.86]. There was no significant time (*t*) effect [F(1,78)=0.051; *p*=0.821; *d*=0.06] and no group (*g*) effect [F(1,78)=2.409; *p*=0.125; *d*=0.35; Table 2], and non significant difference in Pre SαKl levels between CR and ST [F(1,78)=0.033; *p*=0.857; *d*=0.59]. However, a significant difference in Post SαKl levels between the CR and ST groups was observed with a moderate effect size [F(1,78)=8.995; *p*=0.004; *d*=0.7]. Further, in the CR group, a significant (Pre–Post) increase in SαKl levels was produced with a large effect size [F(1,78)=25.432; *p*=0.001; *d*=0.87] while in the ST group, levels dropped slightly, yet not significantly, after exercise and the effect size was small [F(1,78)=1.712; *p*=0.195; *d*=0.37; Figure 3].

**Table 2:** Behavior of SαKl levels over time in the study groups.

SαKl (pg/ml)	Mean±SD		*p*(*t*)	*p*(*g*)	*p*(*txg*)
	Pre	Post			
CR (*n*=43)	982.28±221.6	1172.28±238.14	0.821	0.125	**0.001**
ST (*n*=39)	1058.30±250.16	1001.97±231.78			

Results shown as the mean and standard deviation (mean±SD). Significance set at *p*<0.05.

**Figure 4:**
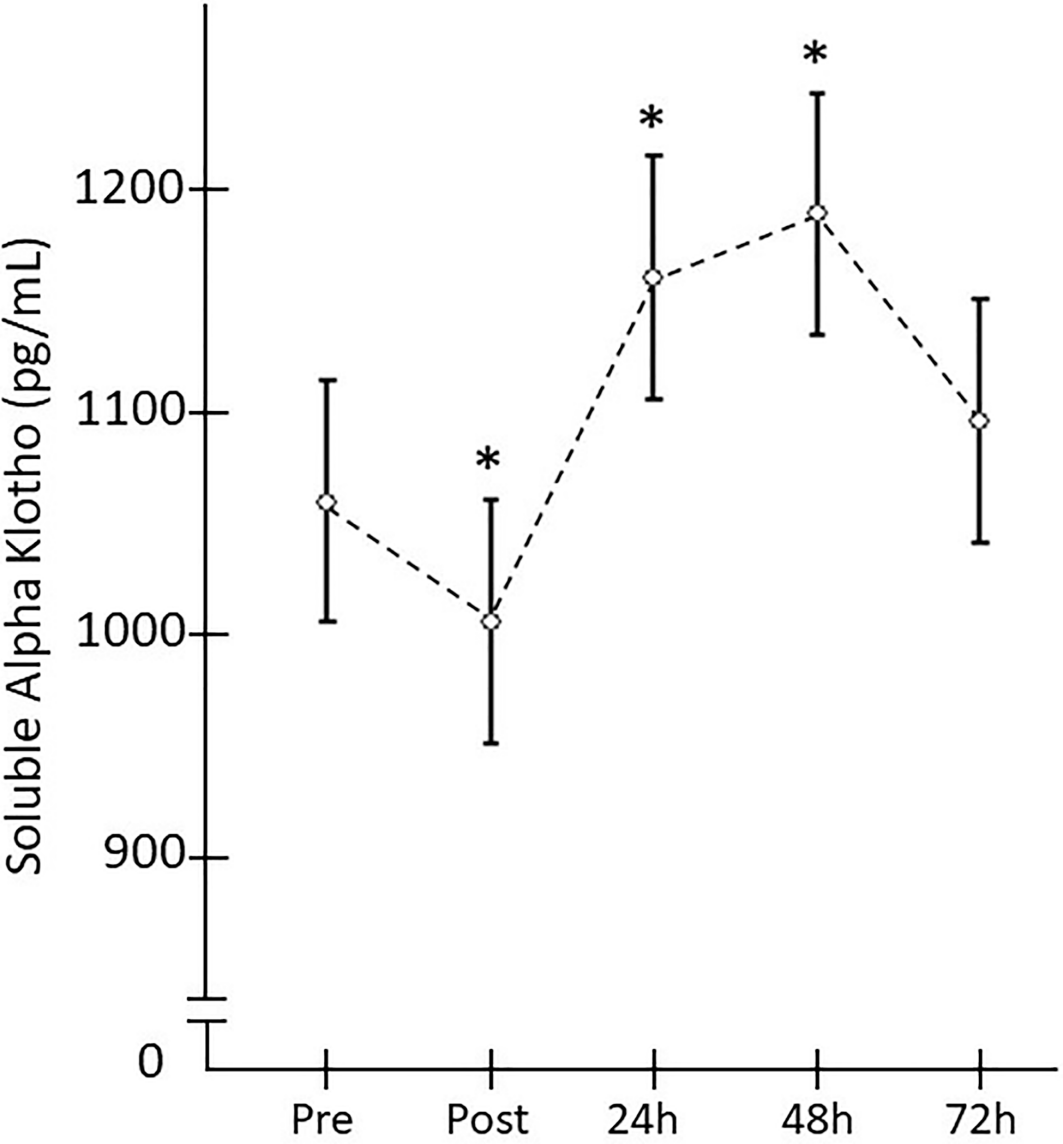
Plasma SαKI levels monitored after the strength exercise intervention. *Significant difference vs. baseline (*p*<0.05).

When the SαKl response to strength exercise was monitored over 72h, significant changes were produced [F(4,152)=4.182; *p*=0.009; *d*=0.663]. Thus, there was a significant decrease Pre- vs. Post-acute ST exercise (*p*=0.025; *d*=0.756) and significant increase between Pre vs. 24h (*p*=0.033; *d*=0.717) and Pre vs. 48h (*p*=0.015; *d*=0.827; Figure 4).

## Discussion

To our knowledge, our study is the first to compare the immediate effects of an acute cardio-respiratory or strength exercise session on plasma SαKl levels, despite several randomized controlled trials of the klotho response to acute cardiorespiratory exercise (Saghiv et al., 2015a; Santos-Dias et al., 2017; Tan et al., 2018). Further, the literature contains only descriptive studies of this response to strength training as the main exercise component (i.e., weightlifters; long-distance runners vs. sprinters; Saghiv et al., 2017a,b) or studies correlating protein levels with limb strength (Semba et al., 2012, 2015; Saghiv et al., 2015a).

Our study is also the first to analyze the time course of plasma SαKl after strength exercise. A significant increase in protein levels has been described immediately after the end of an acute cardiorespiratory exercise session (Saghiv et al., 2015a,b; Santos-Dias et al., 2017), and a recent study has shown that levels remain elevated compared to baseline up to 7days after the intervention (Tan et al., 2018). In contrast, only one study conducted in a mouse model has examined SαKl levels over the days following acute strength exercise (Welc et al., 2020).

Our results indicate that in healthy active men, plasma SαKl levels increase in response to acute cardiorespiratory exercise but decrease immediately after the end of acute strength exercise, and then rise to above pre-exercise levels within 24h. The effect of acute exercise on SαKl levels in healthy active men has been scarcely addressed. Some authors have reported comparable results to those observed in our CR group for a similar exercise intensity and participant age (Saghiv et al., 2015a; Santos-Dias et al., 2017; Tan et al., 2018). Our results are also consistent with reports of a significant immediate increase in SαKl levels after a session of predominantly cardiorespiratory exercise (Saghiv et al., 2015a,b; Santos-Dias et al., 2017). Moreover, increased SαKl levels have also been observed after cardiorespiratory exercise programs lasting from 12 to 16weeks in young people and those over 60years of age (Avin et al., 2014; Matsubara et al., 2014).

As so far only descriptive studies have been carried out in humans, the effect of strength exercise on SαKl levels remains unclear. Significantly lower levels of SαKl have been reported in weightlifters and sprinters compared to inactive subjects or athletes practicing other disciplines (Saghiv et al., 2015a, 2017). In contrast, in older adults (>65years), protein levels have been positively correlated with upper limb strength (Semba et al., 2012) but not with lower limb strength (Semba et al., 2015). In our ST group, we observed a decrease in SαKl values at the end of the acute exercise session contrary to that found in the CR group. The only literature we found to be in accord with these results is an intervention comparable to a strength training bout of exercise in a mouse model, in which SαKl levels were noted to fall after exercise (Welc et al., 2020). Further studies are needed in humans to confirm that an acute session of strength exercise causes an immediate decrease in SαKl levels.

It has been well-established that SαKl levels remain elevated in healthy individuals aged 44–51years for at least 7days after acute high-intensity cardiorespiratory exercise (Tan et al., 2018). Interestingly, it has also been shown in mice SαKl levels gradually recover following a strength exercise session (Welc et al., 2020). These last authors described a similar behavior pattern of the protein in a murine model as observed here. Thus, it seems that SαKl synthesis is inhibited or slowed down after strength exercise, yet induced after 24h. Again, further studies in humans are needed to confirm the behavior over time of SαKl protein after a strength training session. It should be considered that SαKl is intracellularly secreted in muscle fiber cells among others (Kuro-o et al., 1997; Avin et al., 2014). Avin et al. (2014) proposed cross-talk between skeletal muscle activity and SαKl expression to gain strength, whereby biochemical events originating within the muscle, such as ROS regulation, could be important determinants for the regulation of SαKl itself. Thus, some authors propose that the participation of SαKl could be adjusted depending on the molecular demands provoked by the exercise stimulus (Egan and Zierath, 2013; Daneshgar and Dai, 2020).

**Figure 5:**
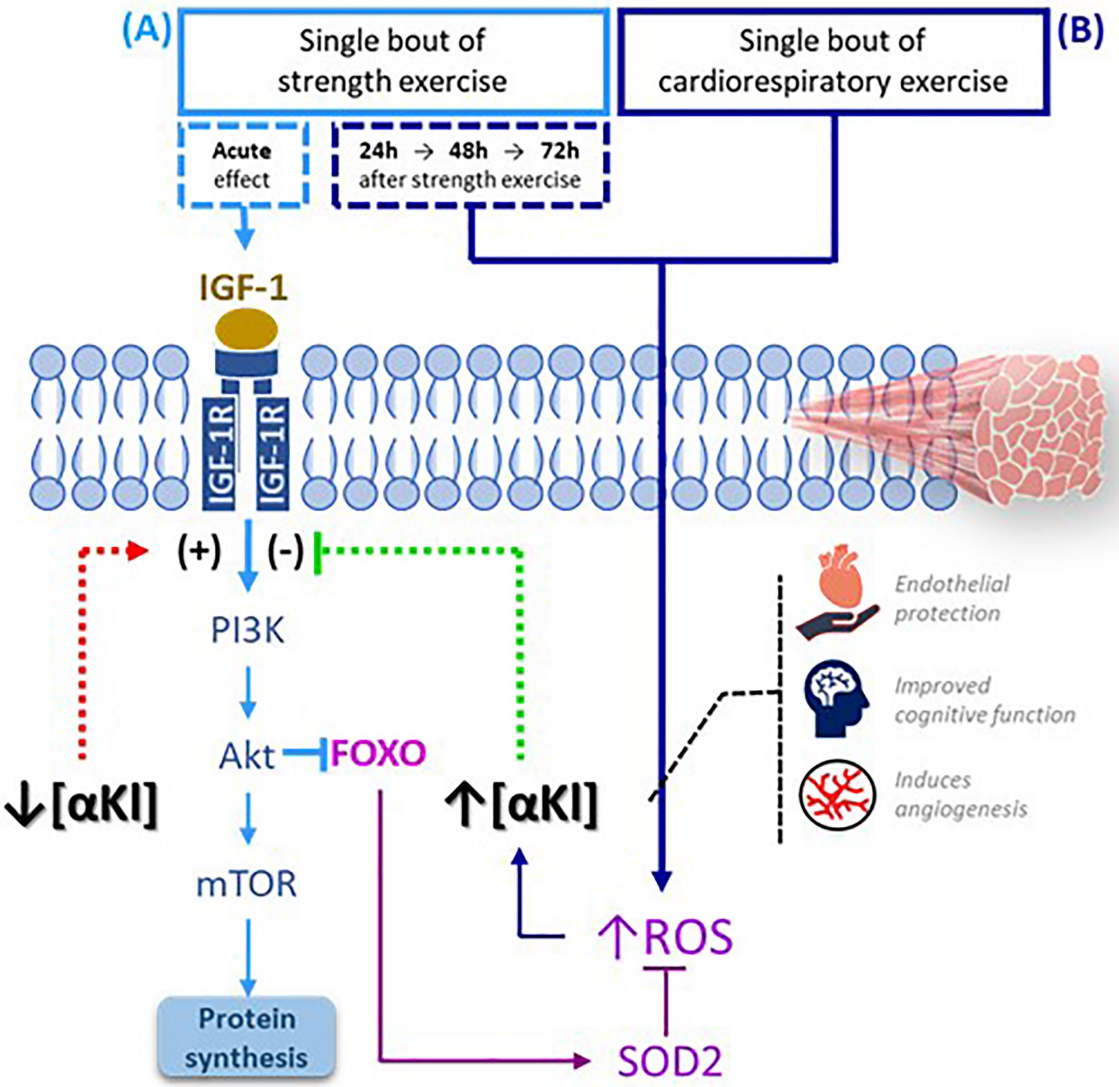
SαKI as a regulator of exercise-activated molecular signaling pathways. **(A)** Following a bout of strength exercise, plasma SαKI levels fall so that the signaling pathway PI3K-Akt-mTOR is activated through IGF-1 induction with the main objective of protein synthesis. **(B)** Plasma SαKI levels rise in response to elevated ROS produced by the exercise stimulus just after completing the session in the case of cardiorespiratory exercise and starting 24h after its completion in the case of strength exercise. ROS levels are regulated through the activation by FOXO of enzymes such as SOD2. FOXO: Forkhead box O; IGF-1: insulin growth factor type 1; IGF-1R: insulin growth factor type 1 receptor; ROS: reactive oxygen species; PI3K: phosphoinositide 3-kinase; Akt: serine/threonine kinase; mTOR: target mechanism of rapamycin kinase.

A session of exercise whose main component is cardiorespiratory prompts an immediate increase in mitochondrial metabolism, which triggers the rapid buildup of ROS (Egan and Zierath, 2013). A plausible hypothesis is that the significant accumulation of the protein is produced just before the end of a bout cardiorespiratory exercise in an effort to regulate these ROS levels. On the other hand, we should also consider that the controlled muscle damage produced by the eccentric component of strength training maintains, during the initial stages sufficient levels of ROS to activate protein metabolism (Egan and Zierath, 2013). In view of this observation, it could be that in this type of exercise, low levels of SαKl are maintained to stimulate the protein repair mechanism and do not increase until there is a significant accumulation of ROS (Papaconstantinou, 2009; Figure 5). Nevertheless, additional basic science studies are needed to verify these hypotheses.

Knowledge of the SαKl response to exercise is of interest, as this tool is being ever more used in clinical practice. The increase in SαKl induced by cardiorespiratory exercise coincides with a beneficial effect of this type of exercise on the cardiovascular system, and with the protective role of SαKl against cardiovascular disease in general and endothelial damage in particular (Matsubara et al., 2014). In addition, strength training with an eccentric component helps to combat the loss of muscle mass and functionality in general and may improve the course of disease (Hody et al., 2019). Besides, this type of exercise generates adaptations that also benefit the immune system. Muscle is considered as an immunogenic organ. When a muscle contracts, certain pro-inflammatory or anti-inflammatory interleukins are released, which are responsible for cell communication between the different organs and tissues through the bloodstream depending on the metabolic demands (Coffey and Hawley, 2007). Hence, knowing the acute effects of different exercise modalities on plasma αKl concentrations, and that strength exercise leads to an increase in its levels after 24h may help us to develop exercise strategies designed to improve the levels of this protein, which is now emerging as a marker of a good prognosis for some diseases (Kuro-o, 2019).

Our study has several limitations. According to a theoretical model of oxidative stress as a possible explanation for the behavior of SαKl levels, it would be of interest to consider other biomarkers such as SOD or malondialdehyde to better understand these responses to both types of exercise. Further, as we stated above, it is well-documented that plyometric exercise often results in significantly muscle damage (Miyama and Nosaka, 2004; Howatson et al., 2012). However, an important limitation of our study is that no muscle damage or muscle fatigue markers (e.g., delayed onset muscle soreness, creatine kinase, and muscle function) for none of CR and ST groups were included in the study. Moreover, the male only study design is a limitation to be able to generalize the results. Future studies that include both men and women would be necessary to see if there are differences in the response to SαKl between the sexes. Another limitation of the present study, stands in the differences in the time points of the blood sample analysis between CR and ST group. SaKl were only measured at baseline and immediately after session for the CR group, while it were measured at baseline and immediately after the session, as well as 1, 2, and 3days after session for the ST group.

In conclusion, the effect of an acute exercise intervention on SαKl levels in healthy physically active men seems to depend on the type of exercise executed. In our study, this determined that plasma levels of the klotho protein increased after acute cardiorespiratory exercise yet diminished immediately after an acute strength exercise session, only to increase with respect to pre-exercise levels 24h later.

## Data Availability Statement

The original contributions presented in the study are included in the article/supplementary material, further inquiries can be directed to the corresponding author.

## Ethics Statement

The studies involving human participants were reviewed and approved by the Ethics Committee of the Community of Madrid (CEIm-R. Reference number: 47/764390.9/17). The patients/participants provided their written informed consent to participate in this study.

## Author Contributions

TI and TY prepared the first draft of the manuscript. TI, IS-L, DD-B, LS-B, and VF-E recruited the sample and collected data. TI and ID-V analyzed data. CS, ML, and MP-R conceived and designed the study. CS and MP-R initiated the overall project. All authors made substantial contributions to revision of the document prior to submission. All authors read and approved the final version of the manuscript.

## Conflict of Interest

The authors declare that the research was conducted in the absence of any commercial or financial relationships that could be construed as a potential conflict of interest.

## Publisher’s Note

All claims expressed in this article are solely those of the authors and do not necessarily represent those of their affiliated organizations, or those of the publisher, the editors and the reviewers. Any product that may be evaluated in this article, or claim that may be made by its manufacturer, is not guaranteed or endorsed by the publisher.
